# Interactions between Connected Half-Sarcomeres Produce Emergent Mechanical Behavior in a Mathematical Model of Muscle

**DOI:** 10.1371/journal.pcbi.1000560

**Published:** 2009-11-13

**Authors:** Kenneth S. Campbell

**Affiliations:** Department of Physiology and Center for Muscle Biology, University of Kentucky, Lexington, Kentucky, United States of America; University of California San Francisco, United States of America

## Abstract

Most reductionist theories of muscle attribute a fiber's mechanical properties to the scaled behavior of a single half-sarcomere. Mathematical models of this type can explain many of the known mechanical properties of muscle but have to incorporate a passive mechanical component that becomes ∼300% stiffer in activating conditions to reproduce the force response elicited by stretching a fast mammalian muscle fiber. The available experimental data suggests that titin filaments, which are the mostly likely source of the passive component, become at most ∼30% stiffer in saturating Ca^2+^ solutions. The work described in this manuscript used computer modeling to test an alternative systems theory that attributes the stretch response of a mammalian fiber to the composite behavior of a collection of half-sarcomeres. The principal finding was that the stretch response of a chemically permeabilized rabbit psoas fiber could be reproduced with a framework consisting of 300 half-sarcomeres arranged in 6 parallel myofibrils without requiring titin filaments to stiffen in activating solutions. Ablation of inter-myofibrillar links in the computer simulations lowered isometric force values and lowered energy absorption during a stretch. This computed behavior mimics effects previously observed in experiments using muscles from desmin-deficient mice in which the connections between Z-disks in adjacent myofibrils are presumably compromised. The current simulations suggest that muscle fibers exhibit emergent properties that reflect interactions between half-sarcomeres and are not properties of a single half-sarcomere in isolation. It is therefore likely that full quantitative understanding of a fiber's mechanical properties requires detailed analysis of a complete fiber system and cannot be achieved by focusing solely on the properties of a single half-sarcomere.

## Author Summary

Quantitative muscle biophysics has been dominated for the last 60 years by reductionist theories that try to explain the mechanical properties of an entire muscle fiber as the scaled behavior of a single half-sarcomere (typical muscle fibers contain ∼10^6^ such structures). This work tests the hypothesis that a fiber's mechanical properties are irreducible, meaning that the fiber exhibits more complex behavior than the half-sarcomeres do. The key finding is that a system composed of many interacting half-sarcomeres has mechanical properties that are very different from that of a single half-sarcomere. This conclusion is based on the results of extensive computer modeling that reproduces the mechanical behavior of a fast mammalian muscle fiber during an imposed stretch without requiring that titin filaments become more than 3-fold stiffer in an activated muscle. This work is significant because it shows that it is probably not sufficient to attribute functional properties of whole muscle fibers solely to the behavior of a single half-sarcomere. Systems-level approaches are therefore likely to be required to explain how known structural and biochemical heterogeneities influence function in normal and diseased muscle tissue.

## Introduction

Many biological systems are irreducible meaning that they have more complicated properties than the structures of which they are composed. Detailed understanding of a complete system therefore requires knowledge both about how its individual components function and about how those components interact. A property of the complete system is described as emergent if it arises because of interactions between components and is not a property of a single component in isolation. Studying the emergence of new properties is an important aspect of modern systems biology and the approach has produced important new insights into many living systems [1].

Although systems-based models of muscle are now being developed [2], alternative reductionist models have dominated quantitative muscle biophysics for the last 60 years. The main strategy has been to try and explain the properties of an entire muscle fiber as the scaled behavior of a single half-sarcomere. This technique was pioneered by A.F.Huxley in 1957 [3] and it has been outstandingly successful. For example, reductionist half-sarcomere theories can explain virtually all of the mechanical effects that occur immediately after a muscle fiber is subjected to a very rapid length or tension perturbation [4],[5].

Muscle fibers do however exhibit some mechanical properties that are not immediately consistent with the expected behavior of a single half-sarcomere. The goal of the present work was to determine whether one specific experimental effect might be an emergent property of a group of half-sarcomeres as opposed to an inherent property of a single one. The analysis focused on the tension responses produced by stretching a chemically permeabilized rabbit psoas muscle fiber. If this type of preparation is stretched when it is inactive, the force response is relatively small and probably largely attributable to the elongation of titin molecules [6]. When the preparation is activated and then lengthened, the stretch response contains an additional, larger, component reflecting the displacement of populations of attached cross-bridges away from the distributions that they adopted during the isometric phase of the contraction. If the filaments keep moving at the same rate for a sufficiently long time, the standard mathematical theories (for example, [3]) predict that cross-bridge populations will reach new steady-state distributions dictated by the strain-dependence of the myosin rate transitions and the velocity of the imposed length change [7]. If steady-state is indeed achieved, the cross-bridge population distributions will remain stable and the force due to attached cross-bridges will therefore remain constant. This simple analysis implies that titin molecules are the only molecular structures inside the half-sarcomere that can produce a force that increases during the latter stages of an imposed stretch. If titin behaves as an elastic spring that is independent of the level of Ca^2+^ activation the rate at which force rises late in an imposed stretch should therefore be the same in maximally-activated fibers as it is in relaxed fibers. In fact force rises >3-fold faster in activated rabbit psoas fibers than it does in the same fibers when they are inactive [7].

One possible explanation for this effect is that the properties of molecules within each half-sarcomere change when a muscle is stretched while it is activated. For example, titin filaments could become stiffer, or the cross-bridge populations could fail to reach steady-state during a prolonged movement. Both of these effects could potentially reflect force-dependent protein-protein interactions [8]. A second possible explanation is that the half-sarcomeres continue to operate as they did before the stretch and that the measured experimental behavior is an emergent property of a collection of heterogeneous half-sarcomeres. These explanations are not mutually exclusive so it is also possible that both effects contribute to the activation dependence of the latter stages of the stretch response. An argument against variable titin properties being the sole explanation is that the magnitude of the Ca^2+^-dependent stiffening required to explain the behavior observed in psoas fibers (∼300% increase in titin stiffness) is much larger than that (∼30% increase in stiffness) observed in experiments that have specifically investigated titin's Ca^2+^-sensitivity [9]. The idea that the activation-dependence of the latter stages of the stretch response could reflect emergent behavior of a collection of half-sarcomeres might be inferred from a number of previous reports [10]–[12] but it does not seem to have been explicitly stated or analyzed in quantitative detail before.

This paper presents a mathematical model that was developed to investigate the potential emergence of new mechanical behavior in a system composed of multiple half-sarcomeres. Detailed computer simulations show that the model can reproduce the activation dependence of the latter stages of the stretch response without requiring that titin filaments stiffen when the Ca^2+^ concentration rises. The stretch response of a fast mammalian muscle fiber may therefore be an irreducible property of the complete cell.

## Results

**Figure 1:**
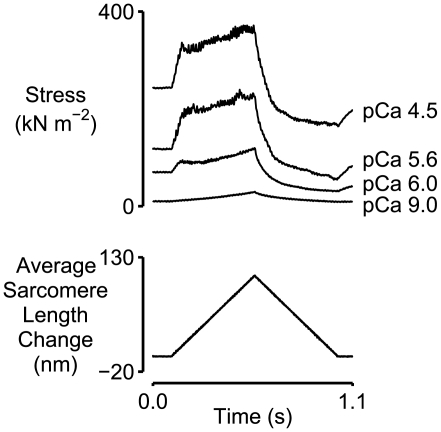
Superposed force and sarcomere length records for a chemically permeabilized rabbit psoas fiber. The fiber was subjected to a ramp stretch followed by a ramp shortening in 4 different pCa solutions. Force rises more quickly during the latter stages of the stretch when the fiber is activated. The records in the pCa 9.0 and pCa 4.5 solutions are re-plotted from Fig 4 of Campbell & Moss [7].

Fig 1 shows experimental force records for a chemically permeabilized rabbit psoas fiber subjected to a ramp lengthening followed by a ramp shortening in four different pCa solutions. The rate at which force rose during the latter stages of the stretch increased with the level of Ca^2+^ activation. Data from 5 fibers showed that the slope (estimated by linear regression) of the tension response during the last one-third of the stretch was 3.26±0.87 (SD) times greater (t-test for value greater than unity, p<0.001) in pCa ( = −log_10_[Ca^2+^]) 4.5 solution (maximal Ca^2+^ activation) than it was in pCa 9.0 solution (minimal Ca^2+^ activation). As discussed in the Introduction, the increased slope in the pCa 4.5 condition is not consistent with the expected behavior of a single population of cycling cross-bridges arranged in parallel with an elastic component that has properties that are independent of the level of activation.

**Figure 2:**
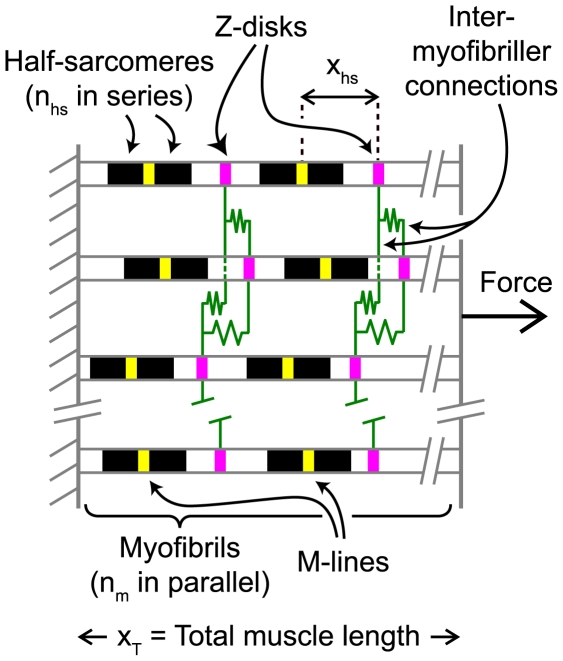
Model Framework. Half-sarcomeres were connected in series to form myofibrils. Myofibrils were arranged in parallel to form larger frameworks. Z-lines at the end of every second half-sarcomere were linked to the corresponding Z-lines in all of the other myofibrils by linear elastic links of stiffness *k*_im_. Half-sarcomeres were represented mathematically as a population of cycling cross-bridges and an elastic element arranged in parallel. The smallest framework considered in this work contained a single half-sarcomere. The largest framework had 50 half-sarcomeres in each myofibril and 6 myofibrils.

**Figure 3:**
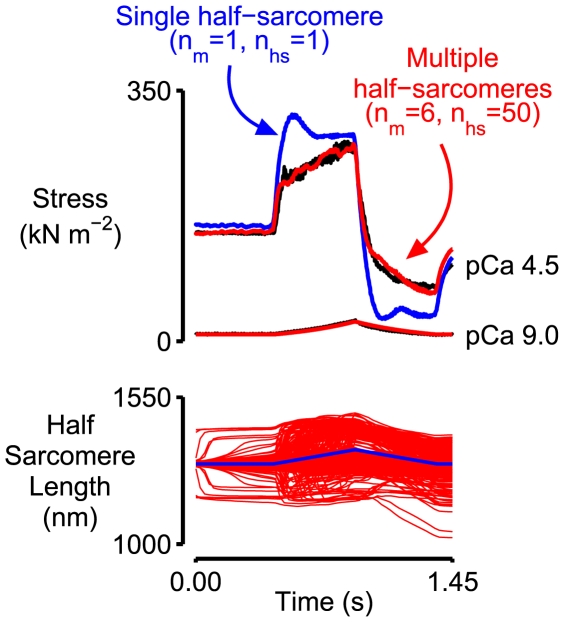
Simulated force and half-sarcomere length traces for the single and multi-half-sarcomere frameworks. Top panel. The black lines in the top panel show the experimental force records for the pCa 9.0 and pCa 4.5 conditions from Fig 1. The red lines show the best-fitting force records for the framework containing 300 half-sarcomeres. The model parameters for this simulation are listed in Tables 1 and 2 (Column 3 in both cases). The blue lines show the simulated force records for a framework with a single half-sarcomere and otherwise identical parameters. Bottom panel. Half-sarcomere length traces for the multi-half-sarcomere (red) and single-half-sarcomere (blue) frameworks.

Computer simulations were performed to test the hypothesis that the activation dependence of the latter stages of the force response may be an emergent property of a collection of half-sarcomeres. The model is summarized in Fig 2 and explained in detail in Materials and Methods. Parameters defining the passive mechanical properties of the half-sarcomeres (Table 1, Column 3) were determined by fitting Eq 8 to an experimental record measured in pCa 9.0 solution. Multidimensional optimization procedures were then used to adjust the other parameters defining the model's behavior in an attempt to fit the simulated force response to the experimental record measured in pCa 4.5 solution. The best-fitting force response obtained in this manner is shown in red in the top panel in Fig 3. The corresponding model parameters are listed in Table 2 (Column 3).

**Table 1:** Parameter values defining passive mechanical properties.

Description	Parameter	Numerical Value			Units
		Rabbit Psoas^1^	Rabbit Psoas^2^	Rat Soleus^3^	
Non-linear Passive Component^4^	σ	4800		5710	N m^−2^
	*x*_offset_	1270		1350	nm
	*L*	49.7		36.0	nm
Linear Passive Component^5^	*k*_pas_		309		N m^−2^ nm^−1^
	*x*_slack_		1270		nm

**Figure 4:**
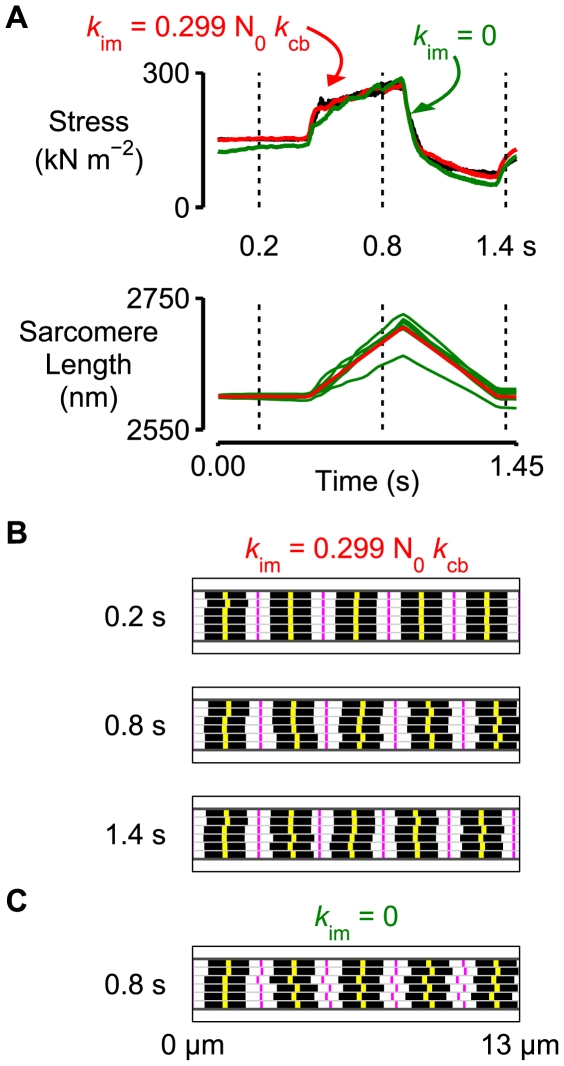
The mechanical properties of the multi-half-sarcomere framework depend on the stiffness of the inter-myofibrillar links. A) The black line in the top panel shows the experimental force record for the pCa 4.5 condition from Fig 1. The red lines show the force and mean sarcomere length (averaged perpendicular to the filaments) records for the multi-half-sarcomere simulations from Fig 3. The green lines show simulations performed with a model that is identical with the sole exception of *k*_im_ which is equal to zero. B) Computer-rendered striation patterns for the simulations drawn in red in Panel A with finite *k*_im_. Thick filaments are drawn in black. M-lines are yellow. Z-disks are magenta. The Z-disks are all in register but the M-lines are sometimes misaligned. Only ∼1/5 of the length of the framework is shown in the diagram for clarity. C) As for Panel B but drawn for the simulations with *k*_im_ equal to zero. The Z-disks are also misaligned. A movie showing how the striation patterns change during the length perturbation is included as Supporting Information (Video S1).

**Figure 5:**
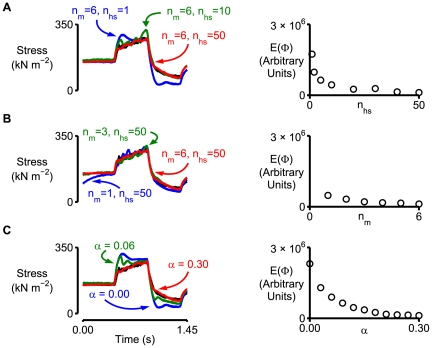
Effects of changing key model parameters. Left hand panels show the experimental force record for the pCa 4.5 condition from Fig 1 in black and calculated force records from selected simulations in other colors. Right-hand panels show values of E(Φ) (Eq 10) for different simulations. Lower values of E(Φ) indicate that the simulated record fits the experimental data better. Unless otherwise stated, all simulations were performed using the model parameters listed in Tables 1 and 2 (Column 3 in both cases) and a model framework with *n*_m_ = 6 and *n*_hs_ = 50. A) Effect of increasing the number of half-sarcomeres (*n*_hs_) in each myofibril. B) Effect of changing the number of myofibrils (*n*_m_). C) Effect of changing the variance of the number of myosin heads per half-sarcomere (α, Eq 7).

1 See Figs 3, 4 and 5.

2 See Fig 6A.

3 See Fig 6B.

4 Equation 8.

5 Equation 9.

All values are shown to 3 significant figures.

**Table 2:** Parameter values determined by multi-dimensional optimization.

Description	Parameter	Numerical Value			Units
		Rabbit Psoas (Non-linear Passive Component)^1^	Rabbit Psoas (Linear Passive Component)^2^	Rat Soleus (Non-linear Passive Component)^3^	
Myosin power-stroke^4^	*x*_ps_	9.52	9.77	10.1	nm
Parameters related to rate functions^5^	*f*_1,max_	3.58	4.02	0.633	s^−1^
	*f*_1,falloff_	0.0244	0.0299	0.169	nm^−1^
	*f*_2,base_	0.298	0.291	22.5	s^−1^
	*f*_2,gradient_	−0.920	−1.10	0	s^−1^ nm^−1^
	*f*_2,intercept_	10.3	23.4	0	s^−1^
	*f*_2,max_	6.97	5.26	11.9	s^−1^
	*f*_3,base_	7.51	6.44	9.37	s^−1^
	*f*_3,pos_	1.43	1.40	1.27	nm
	*f*_3,neg_	3.94	3.78	0.521	nm
	*α*	0.30	0.33	0.17	Dimensionless
Binding Energies^6^	*A*_1,base_	−4.77	−4.73	−6.24	*k*_B_ T
	*A*_2,base_	−19.9	−19.9	−17.5	*k*_B_ T
Inter-myofibril link stiffness^7^	*k*_im_	0.299 N_o_ *k*_cb_	1.71 N_o_ *k*_cb_	0 N_o_ *k*_cb_	N m^−1^

1 See Figs 3, 4 and 5.

2 See Fig 6A.

3 See Fig 6B.

4 See Fig 7.

5 Equation 5.

6 *k*_B_ is Boltzmann's constant (1.381×10^−23^ J K^−1^). T is 288 K.

7 See Fig 2.

All values are shown to 3 significant figures.

The blue lines in the top panel in Fig 3 show the force responses produced by a single half-sarcomere framework with the same model parameters. The simulated force records for the single and multi-half-sarcomere frameworks are the same for the pCa 9.0 condition (where there are no attached cross-bridges) but different for the pCa 4.5 condition. Note in particular that the multi-half-sarcomere framework predicts a smaller short-range force response and a tension that rises more steeply during the latter stages of the stretch. This progressively increasing tension is not a property of a single activated half-sarcomere in these simulations and therefore reflects interactions that occur between half-sarcomeres; it is an emergent property of the multi-half-sarcomere framework.

The red lines in the bottom panel in Fig 3 show the length traces for the 300 half-sarcomeres in the larger framework superposed. (The traces are shown in more detail in Supporting Information Figure S1.) Although individual half-sarcomeres followed length trajectories defined by Eq 2 the behavior of the overall system is chaotic. During the stretch, for example, some half-sarcomeres are lengthening, some are shortening, and some remain nearly isometric. The behavior of each pair of half-sarcomeres on the other hand is more orderly. Indeed, at any given time-point in the simulation, all the full sarcomeres had virtually the same length. This is because the inter-myofibrillar links (Fig 2) were sufficiently stiff to keep the Z-disks in register during the activation. The effect is demonstrated in Fig 4B where the computer-rendered striation patterns show that the Z-disks (drawn in magenta) are always aligned whereas the M-lines (drawn in yellow) are frequently displaced from the middle of the sarcomere.

Z-disk alignment is no longer maintained in the simulations if the inter-myofibrillar links are ablated *in silico* by setting *k*_im_ equal to zero (Fig 4C). In this situation, mean sarcomere length averaged perpendicular to the filaments for the different half-sarcomere pairs (green lines in Fig 4A) is no longer constant although mean sarcomere length averaged parallel to the filaments is always the same in the different myofibrils. (This has to be the case because all the myofibrils have the same length and contain the same number of sarcomeres.) A movie showing how the computer-generated striation patterns change during the length perturbations is provided as Supporting Information Video S1. Interestingly, the predicted isometric force value is lower for the simulations with *k*_im_ equal to zero. The area under an xy-plot of force against length during the stretch (not shown) is also lower indicating that the framework simulated without inter-myofibrillar links would absorb less energy during an eccentric contraction. This mimics experimental results obtained by Sam *et al.* [13] using muscles from desmin-null mice.

Fig 5 shows the effects of changing the size of the model framework and the numerical value of a key model parameter. All simulations were performed with the parameters listed in the third columns of Tables 1 and 2 except for Fig 5C where α (Eq 7) was varied as shown. Increasing *n*_hs_ (the number of half-sarcomeres in each myofibril) from 1 to 10 in a framework with 6 myofibrils markedly improved the fit to the experimental record. The additional improvement gained by further increasing *n*_hs_ to 50 was more modest. When there were already 50 half-sarcomeres in each myofibril, increasing the number of myofibrils did not dramatically improve the fit during the stretch response (Fig 5B) but it did help to stabilize isometric force before the stretch. This is at least partly because the presence of inter-myofibrillar links stabilized sarcomere (but not half-sarcomere) lengths (Fig 4B and C). The effects of varying α to alter the amount of half-sarcomere heterogeneity in the largest framework are summarized in Fig 5C. Note that increasing α beyond 0.1 did not substantially change the fit to the experimental data and that the simulated response for the framework with 300 half-sarcomeres and α equal to zero was not different from that of the single half-sarcomere framework with the same model parameters. This second point demonstrates that a fiber system does not exhibit emergent properties if the half-sarcomeres of which it is composed are all identical.

This informal sensitivity analysis suggests that the activation dependence of the latter stages of the stretch response is more likely to reflect inhomogeneity between half-sarcomeres along a myofibril than inhomogeneity between different myofibrils. This prediction is based on the computed results shown in Fig 5A and B. Increasing the number of half-sarcomeres from 1 to 50 in a framework with 6 myofibrils markedly changed the slope of the force response during the second half of the stretch (Fig 5A). In contrast, increasing the number of myofibrils in a framework with 50 half-sarcomeres (Fig 5B) reduced the magnitude of oscillations in the computed force records but did not substantially alter the underlying trend of the responses.

**Figure 6:**
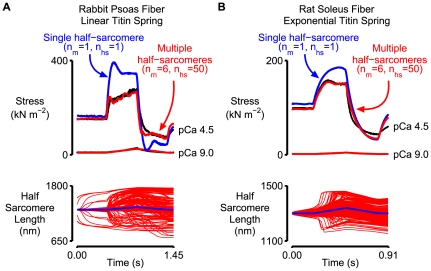
Additional simulations. A) Rabbit psoas data from Fig 1 simulated with a linear passive component (Eq 9). B) Rat soleus data from Fig 4 of Campbell & Moss [14] simulated with a non-linear passive component. The top plots in both panels show experimental force records drawn in black, best-fit force records simulated with a multi-half-sarcomere framework in red and force records simulated using a single half-sarcomere framework and otherwise identical parameters in blue. The bottom panels show half-sarcomere length traces for the multi- and single half-sarcomere frameworks in red and blue respectively. Model parameters for both sets of simulations are listed in Tables 1 and 2.

The value of the parameters defining *F*_pas_ (Table 1, Column 3) were determined by fitting Eq 8 to force records measured for a fiber in pCa 9.0 solution during small dynamic stretches (4% muscle length) imposed from a starting sarcomere length of ∼2600 nm. It is therefore possible that the calculated parameters overestimate the isometric passive tension that would have been measured if the half-sarcomeres were stretched more than 4%. (The passive length tension relationship was not measured in the original experiments [7] so the relevant experimental data were not available for comparison.) To eliminate any possibility that the tension response during the latter stages of an imposed stretch is only activation-dependent in the current simulations because the titin filaments are unrealistically stiff at long lengths, additional calculations were performed with a linear passive component. The parameters defining *F*_pas_ in this case (Table 1, Column 4) were determined by fitting Eq 9 to the same pCa 9.0 force record. Passive force calculated in this way did not reach the maximal Ca^2+^-activated value until the sarcomeres were stretched beyond 3500 nm. The best-fitting force simulations deduced by multi-dimensional optimization with the linear titin component are shown in red in Fig 6A. While the simulation of the active fiber does not match the experimental data as well as the simulations (Fig 3) performed with the non-linear titin component (r^2^ = 0.93 as opposed to r^2^ = 0.98) it does reproduce the activation-dependence of the slope of the force response during the latter stages of the stretch.

Rat soleus fibers exhibit a stretch response that is qualitatively different from that produced by rabbit psoas fibers [14]. Instead of force rising during the latter stages of the movement, force tends to peak and then fall slightly to a plateau that is maintained as long as the stretch persists. (A similar plateau is observed when frog tibialis anterior fibers are stretched [15]). Although the shape of the response seems to imply that passive titin properties are less important in rat soleus fibers than they are in rabbit psoas fibers, Campbell & Moss [14] showed that a single half-sarcomere model produced the best-fit to the real Ca^2+^-activated data when the cross-bridges were arranged in parallel with a titin spring that was ∼3 times stiffer than that measured experimentally in pCa 9.0 solution. The behavior of the soleus fibers was thus very similar to that described here for psoas preparations. This suggests that simulations performed with a multi-half-sarcomere framework might also produce a better fit to the mechanical data from soleus fibers than a model based on a single half-sarcomere. Fig 6B shows the results of calculations performed to test this hypothesis. Parameter values for the simulations are listed in Tables 1 and 2 (Column 5 in both cases). The predictions for the multi-half-sarcomere framework fit the experimental data well (r^2^ = 0.97) and, as in the case of the simulations of psoas fiber data, predict a lower isometric force and a less prominent short-range response than the simulations performed with a single half-sarcomere framework and otherwise identical model parameters.

## Discussion

The main goal of this work was to test the hypothesis that the activation dependence of the latter stages of the stretch response of a fast mammalian muscle fiber may be an emergent property of a group of half-sarcomeres. The simulations presented in Fig 3 show that the hypothesis might be correct. Even though the passive mechanical properties of the individual half-sarcomeres are invariant, the force produced by the multi-half-sarcomere framework rises progressively during the latter stages of the stretch whereas the force produced by the single half-sarcomere model remains almost constant after the muscle's short-range response. This means that the simulations performed with the multi-half-sarcomere framework fit the experimental data better and they do so without requiring that the titin filaments become stiffer in the activating condition.

While the simulations presented in this work suggest that the activation dependence of the latter stages of the stretch response could be an emergent behavior of the muscle fiber, they do not prove that it is. Reproducing an experimental result with a computational model is not the same thing as demonstrating a mechanistic effect in an actual experiment. Although it would be useful to have a definitive physical test for emergent behavior in muscle fibers, it may not be easy to develop one. For example, it is very difficult to make definitive measurements of titin's Ca^2+^-sensitivity [9] in the absence of potential cross-bridge binding. While myosin ATP-ase inhibitors, such as 2,3-butanedione 2-monoxime [16] and blebbistatin [17], and techniques such as chemical extraction of troponin C [18], reduce active force in skeletal muscles it is not clear that they completely abolish all cross-bridge links without perturbing other structures. Titin's complicated non-linear behavior also means that it would be difficult to infer its mechanical properties in Ca^2+^-activating solutions at physiological sarcomere lengths from measurements made when the sarcomeres were stretched beyond filament overlap to prevent myosin binding. This makes it difficult to measure the properties of titin filaments immersed in Ca^2+^-activating solutions without the experimental results potentially being compromised by a small number of actin-myosin links.

It is also not yet possible to determine that cross-bridge populations do actually reach steady-state during perfectly uniform stretches so transient kinetic effects could contribute to the observed activation-dependent behavior. One intriguing possibility would be to test for the emergence of new mechanical properties in experiments utilizing myofibrillar preparations with different numbers of half-sarcomeres. These experiments would be technically demanding (and probably subject to their own limitations) but a recent report of measurements on a single sarcomere [19] suggests that they may be feasible in the near future.

### Half-sarcomere uniformity

This work provides important new insights and introduces novel simulation techniques but the idea that the mechanical properties of a muscle fiber might be influenced by individual half-sarcomeres behaving in different ways is not new [15], [20]–[22]. One of the controversies in the field is whether sarcomeres ‘pop’, that is, extend rapidly to beyond filament overlap [12]. This behavior can be predicted from an analysis of the steady-state active and passive length tension relationships but it has not been observed in some experiments that have specifically investigated the issue in small myofibrillar preparations [23],[24]. Other data [25] suggest that some sarcomeres in a sub-maximally activated myofibril ‘yield’ and others ‘resist’ during a stretch. The present simulations suggest that there are at least two mechanisms that may reduce the likelihood of (but perhaps not entirely eliminate) popping under normal physiological conditions.

First, attached cross-bridges in half-sarcomeres that are starting to elongate will be stretched thereby producing increased force. If the total length of the muscle fiber is fixed, other half-sarcomeres in the same myofibril will have to shorten and force will therefore drop in these structures. The changes in the forces produced by cross-bridges in the half-sarcomeres that moved are transient because they will dissipate as the myosin heads progress through their normal cycle. However, while they exist, they act in such a way as to reduce the development of additional heterogeneity. *In vivo*, this effect could be enough to prevent the cell from being structurally damaged before it relaxes at the end of the contraction and passive mechanical properties are able to restore the fiber's prior arrangement.

Second, forces in molecules that link half-sarcomeres will help to preserve sarcomere length uniformity. In the current simulations, some of these molecules are represented mathematically by linear springs that connect Z-disks in adjacent myofibrils. It was particularly interesting to discover that the *in silico* ‘knock-out’ of inter-myofibrillar connections (*k*_im_ = 0, Fig 4) reproduced the functional effects observed in mice from desmin-null mice - lower isometric force and decreased energy absorption during imposed stretches [13].

One of the many interesting features of the second phase of the stretch response of activated muscle fibers is that it can be quite variable. Fig 6, for example, shows that it is markedly different in fast and slow mammalian fibers under very similar experimental conditions. Getz *et al.* [11] observed that differences can also be observed within fast fibers from rabbit psoas muscle. Their manuscript notes that the “continued force rise after the critical stretch was sometimes but not always present in our data”. (It is important to note that the stretches used by Getz *et al.* were up to 25 times faster than the ones simulated in the present work. A slow rise in force during the latter stages of the stretch was always observed in the experiments with psoas fibers that are simulated here [7].) Getz *et al.* suggested that the variable nature of their measured responses might reflect different amounts of half-sarcomere heterogeneity in their preparations. Their conclusion is supported by the present simulations.

Half-sarcomere heterogeneity has also been suggested as a potential explanation for residual force enhancement - the augmented force that persists long after a stretch and hold imposed during a maximal contraction [26]. The current simulations support this hypothesis too because Edman & Tsuchiya [10] showed that the size of the enhancement correlates with the magnitude of the second phase of the force response in the stretch that produces it. However, half-sarcomere heterogeneity may not be the only mechanism responsible for residual force enhancement because Edman & Tsuchiya [10] also showed that there could be a small residual enhancement when the conditioning stretch didn't produce a measurable second phase force response.

### Sarcomere length control experiments

Precise measurements of the mechanical properties of single muscle fibers are often performed using a technique known as sarcomere length control [27],[28]. This is an important experimental approach but it should be made clear that the technique does not eliminate the potential emergence of new properties due to the collective behavior of half-sarcomeres. This is because sarcomere length control dictates the mean sarcomere length in a selected region of the muscle fiber rather than the lengths of the individual half-sarcomeres. It is thus the *in vitro* equivalent of the computer simulations discussed in this work in which *x*_T_, the total length of a defined group of half-sarcomeres, is the controlled variable.

### Implications

Many biologists probably regard it as axiomatic that the properties of a muscle fiber vary along its length. After all, organelles, such as nuclei and mitochondria, are localized structures that are not uniformly ‘smeared’ throughout the cell. There are, of course, other sorts of non-uniformity in muscle cells as well. There is good evidence to suggest, for example, that eye muscle fibers express different myosin isoforms along their length [29] and that sarcomeres near the end of a fiber are shorter than those near the middle [30]. Many quantitative models of muscle on the other hand overlook variability within muscle fibers and attribute the mechanical properties of an experimental preparation to the scaled behavior of a single population of cycling cross-bridge that is sometimes arranged in parallel with a passive mechanical component. These reductionist theories have been outstandingly successful at explaining the behavior observed in some specific experiments [31] but the simulations presented in this work suggest that more realistic multi-scale modeling may be required to fully reproduce the behavior of whole muscle fibers.

Multi-scale modeling may be particularly helpful in studies of muscle disease. It is well known, for example, that muscle function is compromised in muscular dystrophy where the primary defect occurs in a large structural protein [32]. Defects in such proteins will affect the way that forces are transmitted between and around myofibrils which, as shown in Fig 4, may significantly alter a muscle's mechanical behavior. This concept is also supported by experimental data. Shimamoto *et al.* [25] recently showed, for example, that modifying Z-disk structure with antibodies can influence the emergent properties of a myofibrillar preparation by altering the way that half-sarcomeres interact.

Finally, the simulations shown in Fig 5C demonstrate that the relatively small amount of half-sarcomere heterogeneity produced by increasing α from 0.0 to 0.1 dramatically alters the mechanical properties of the muscle framework. Further increases in α produce more half-sarcomere heterogeneity but do not substantially alter the predicted force response. This is a very interesting finding because it implies that the mechanical properties of a muscle that was originally perfectly uniform would change markedly if localized structural and/or proteomic abnormalities developed as a result of a disease process and/or unusual mechanical stress. The mechanical properties of a muscle cell that was already slightly heterogeneous on the other hand would not be substantially altered by additional irregularities. This could be a significant advantage for a living cell that is continually repairing itself and which is potentially subject to damaging stimuli and large external forces. Muscle cells may have evolved to become fault-tolerant systems.

### Summary

The mathematical modeling presented in this work suggests that muscle fibers may exhibit emergent mechanical properties that reflect interactions between half-sarcomeres. If this is indeed the case, systems-level approaches will tbe required to explain how known proteomic and structural heterogeneities influence function in normal and diseased tissue.

## Materials and Methods

### Ethics statement

Animal use was approved by the University of Wisconsin-Madison Institutional Animal Care and Use Committee.

### Experimental measurements

All of the experimental records shown in this work were collected by the author in Dr. Richard Moss's laboratory at the University of Wisconsin-Madison. Full details of the experimental procedures and some of the records have already been published [7],[14]. Animal use was approved by the relevant Institutional Animal Care and Use Committee.

### Mathematical model

The structural framework studied in this work (Fig 2) consisted of *n*_m_ parallel chains of myofibrils, each of which was itself composed of *n*_hs_ half-sarcomeres arranged in series. Every second Z-line was linked to the corresponding Z-line in each of the other myofibrils by a linear elastic spring of stiffness *k*_im_. These connections simulated the mechanical effects of proteins such as desmin that connect myofibrils at Z-disks [33].

The force within each half-sarcomere (*F*_hs_) was the sum of *F*_pas_, a ‘passive’ elastic force due to the mechanical elongation of structural molecules such as titin, and *F*_act_, an ‘active’ force produced by ATP-dependent cross-bridge cycling [34],[35].
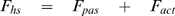
(1)
*F*_pas_ was a single-valued function of the length (*x*_hs_) of each half-sarcomere. *F*_act_ was more complicated and depended on the half-sarcomere's preceding motion. Both force components are described in more detail below.

### Half-sarcomere dynamics

The mechanical behavior of the multi-half-sarcomere framework was simulated by assuming that (1) the force in a given myofibril was the same at every point along its length and (2) the sum of the lengths of the half-sarcomeres in each myofibril was equal to the total muscle length. These assumptions lead to a set of functions
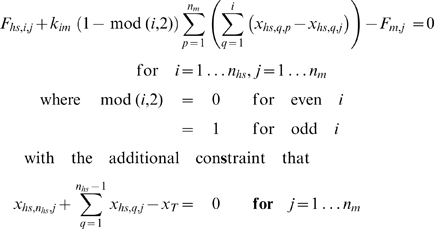
(2)where *F*_hs,i,j_ and *x*_hs,i,j_ respectively describe the force developed by and the length of half-sarcomere *i* in myofibril *j*, *F*_m,j_ is the force in myofibril *j* and *x*_T_ is the total length of the framework (Fig 2). These functions can be solved using a root-finding method (see Numerical Methods section below) to yield the lengths of each half-sarcomere and thus the mechanical state of the framework.

### *F*_act_ forces

**Figure 7:**
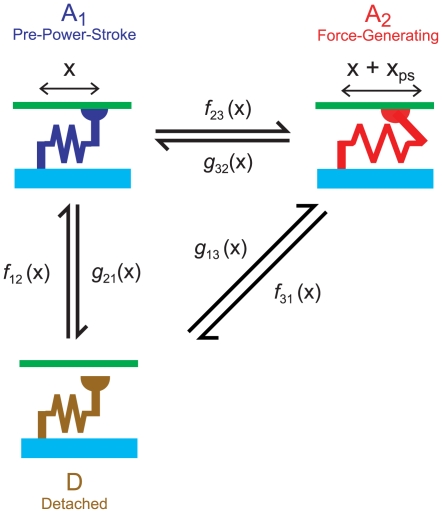
Actin-Myosin kinetic scheme. Cross-bridges cycled through a three state kinetic scheme governed by strain dependent kinetics. The cross-bridge link extended by *x*_ps_ when the cross-bridge made the transition from the A_1_ to the A_2_ state.

*F*_act_ values for each half-sarcomere in the framework were calculated using techniques previously described for a single half-sarcomere model by Campbell & Moss [7]. Myosin heads were assumed to cycle through the 3-state kinetic scheme shown in Fig 7.

**Figure 8:**
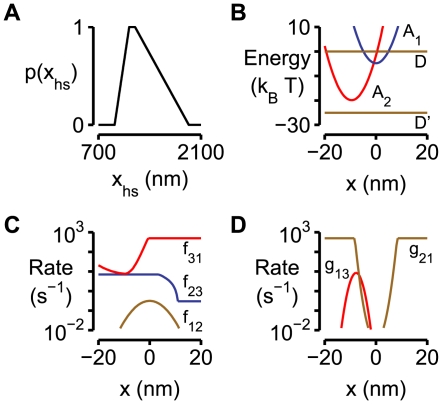
Assumptions underlying the calculations of F_act_ values. A) *p*(*x*_hs_), the proportion of myosin heads that could participate in the cross-bridge cycle in a given half-sarcomere varied as a function of the half-sarcomere length as described in Eq 3. B, C, D) Plots showing the strain-dependence of the (B) free energy diagram (Eq 4), (C) forward rate functions (Eq 5) and (D) reverse rate functions (Eq 6) for the parameter values listed in Table 2 for the simulations of rabbit psoas fiber data with a non-linear passive component.

The proportion *p*(*x*_hs_) of cross-bridges participating in the kinetic scheme in each half-sarcomere was set to zero for all *x*_hs_ during simulations of passive muscle (pCa 9.0 conditions). In simulations of activate muscle (pCa 4.5 conditions), *p*(*x*_hs_) was assumed to scale with the number of myosin heads overlapping the thin filament (Fig 8A) so that
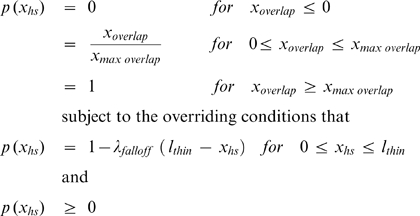
(3)where *x*_overlap_ is *l*_thin_+*l*_thick_−*x*_hs_, *x*_maxoverlap_ is *l*_thick_−*l*_bare_, and *l*_thin_, *l*_thick_, and *l*_bare_ are the lengths of the thin filaments (1120 nm), thick filaments (815 nm) and thick filament bare zone (80 nm) respectively and λ_falloff_ is a model parameter arbitrarily set to 0.005 nm^−1^.

The rate constants defining the probability of a cross-bridge moving to a different biochemical state depended on the length *x* of the cross-bridge link and twelve model parameters (Table 2) that were determined by fitting the simulated force values to representative data records using multidimensional optimization techniques (see below). The spring constant *k*_cb_ for an individual cross-bridge link was defined as 0.0016 N m^−1^ in close agreement with recent experimental estimates for this parameter [36],[37]. Energies for the cross-bridge states (Fig 8B) were defined as
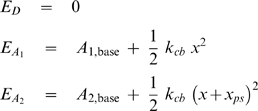
(4)where *x* is the length of the cross-bridge link, *x*_ps_ is the length of the force-generating power-stroke and A_1,base_ and A_2,base_ define the minimum energy of cross-bridge links bound in the A_1_ and A_2_ states respectively. The energy difference between the E_D_ and E_D′_ states (Fig 8B) was 25 *k*_B_T where *k*_B_ is Boltzmann's constant (1.381×10^−23^ J K^−1^) and T was 288 K. (The original experiments were performed at 15°C [7],[37]).

Strain-dependent rate functions *f*_12_(*x*), *f*_23_(*x*) and *f*_31_(*x*) for the forward transitions (Fig 7) were defined as
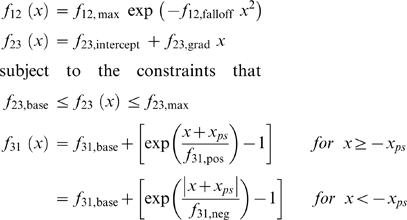
(5)Reverse rate functions *g*_21_(*x*), *g*_32_(*x*) and *g*_13_(*x*) were defined in terms of the forward rate functions and the energy difference between the relevant states [38] as
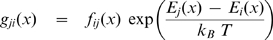
(6)Panels B, C and D in Fig 8 show the strain-dependence of the free energy diagram for the cross-bridge scheme, the forward rate functions and the reverse rate functions used in the simulations shown in Fig 3. The numerical values of the relevant parameters are listed in the third column in Table 2.

The number of myosin heads per unit cross-sectional area in a single half-sarcomere framework was always *N*_0_ (defined in this work as 1.15×10^17^ m^−2^ [36]). Half-sarcomere heterogeneity was incorporated into the simulations of multiple half-sarcomere frameworks by assuming that the number of myosin heads per half-sarcomere was a normally distributed variable. Thus the actual number (*N*_i_) of myosin heads participating in the cross-bridge cycle in half-sarcomere *i* at half-sarcomere length *x*_hs_ was equal to
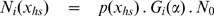
(7)where *G*_i_(α) was a variable randomly selected from a Gaussian distribution with mean of unity and a variance of α.

### *F*_pas_ forces

The passive force *F*_pas_ increased in a non-linear manner as
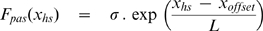
(8)where σ, *x*_offset_ and *L* were determined by curve-fitting [7],[14], with the exception of one set of simulations. Fig 6A shows force records simulated with a passive force that increased linearly with half-sarcomere length as
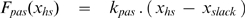
(9)where *k*_pas_ defines the stiffness of the passive elastic spring and *x*_slack_ is the half-sarcomere length at which the spring falls slack.

### Filament compliance

Filament compliance effects [39],[40] were incorporated by assuming that if a half-sarcomere changed length by Δ*x*_hs_ in a given time-step each cross-bridge link in the half-sarcomere changed length by ½Δ*x*_hs_ [11]. This over-simplifies the realignment of actin binding sites and myosin heads that occurs in real muscle fibers but the finite availability of computing power means that it is not yet practical to implement more realistic simulations of filament compliance effects [41]–[44] with a framework containing 300 half-sarcomeres.

### Numerical methods

The mathematical model was implemented as a multi-threaded console application (Visual Studio 2005, Microsoft, Redmond, WA) written in C++. Equation 2 was solved using the newt() function described by Press *et al.* [45] which invokes Newton's method to solve non-linear sets of functions. Δ*x* for cross-bridge populations [7] was set to 0.5 nm. The time-step was set to 1 ms. Reducing these parameters by 50% did not materially change the results of the calculations.

Calculated rate constants (Eqs 5 and 6) were constrained to a maximum value of 500 s^−1^. Rate constants were set to zero if the calculated value was less than 0.01 s^−1^. This simplified the numerical procedures used to solve the evolution of the cross-bridge populations.

Randomly-distributed double-precision numbers were generated using the Mersenne Twister Algorithm [46]. Post-processing of simulation output files and subsequent figure development was performed using custom-written MATLAB (The Mathworks, Nattick, MA) software.

### Parameter optimization

Particle swarm optimization routines [47] were used to fit the force traces predicted by the simulations to selected experimental records. This was done by searching for the lowest attainable value of an error function defined as
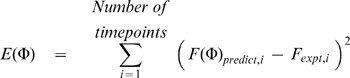
(10)where *F*_expt,i_ is the experimentally-recorded force value at time-point *i* and *F*(Φ)_predict,i_ is the corresponding prediction for parameter set Φ.

Solving Eq 2 for a framework with *n*_m_ = 6 and *n*_hs_ = 50 took ∼0.25 s on a quad-core 2.5 GHz personal computer. Each simulated force response (of order 10^3^ time-steps with 1 ms resolution) therefore required ∼5 minutes to compute. To reduce the wall-time required for the parameter estimation procedures, the calculations were performed using spare screen-saver processing time on ∼30 computers running DEngine (for Distributed computing ENGINE) software developed by the author (http://www.dengine.org). This arrangement allowed typical optimization tasks to be completed using a particle swarm algorithm [47] in ∼2 days (∼10 times faster than if the task was performed using a single representative machine).

## Supporting Information

Figure S1 Half-sarcomeres in different myofibrils do not behave the same way. Data from the simulations for the multi-half-sarcomere framework shown in Fig 3 replotted to show the half-sarcomere length traces in each myofibril. Left-hand panels show the traces for the 50 half-sarcomeres in each myofibril in different colors. Right-hand panels show histograms for the relative number of cross-bridges in each half-sarcomere for the 300 half-sarcomeres in the framework (blue bars, same data in each plot) and the 50 half-sarcomeres in the specified myofibril (red bars).
(3.11 MB EPS)

Video S1 Computer-rendered striation patterns. The movie shows computer-rendered striation patterns for the simulations shown in Fig 4 with and without inter-myofibrillar connections.
(2.08 MB MOV)
